# Potential circadian rhythm-related pathogenic genes in diabetic nephropathy: a multi-omics Mendelian randomization study

**DOI:** 10.1080/0886022X.2026.2663248

**Published:** 2026-05-24

**Authors:** Yanwen Mao, Minghao Zhang, Xiaojuan Li, Zijuan Zhang, Wenhui Rong, Xiaowei Zhang, Long Feng, Jiangyan Xu

**Affiliations:** ^a^ Academy of Medical, Henan University of Chinese Medicine, Zhengzhou, Henan, China; ^b^ Academy of Chinese Medical Sciences, Henan University of Chinese Medicine, Zhengzhou, Henan, China

**Keywords:** Diabetic nephropathy, circadian rhythm, multi-omics, Mendelian randomization analysis, colocalization

## Abstract

This study aims to identify pathogenic genes involved in diabetic nephropathy (DN) through multi-omics analysis of circadian rhythm disruptions to uncover targets for precision medicine. We conducted summary-data-based Mendelian Randomization (SMR) and colocalization analyses using multi-omics quantitative trait loci (QTL) data (methylation quantitative trait loci [mQTL] , expression quantitative trait loci [eQTL], protein quantitative trait loci [pQTL]) to explore genetic associations between circadian rhythm-related genes and DN. Genome-wide association study (GWAS) summary statistics were sourced from major consortia (discovery) and FinnGen (replication). Key gene expressions were validated in GEO (GSE96804). Transcription factors were predicted, and potential drugs (*via* Enrichr) were assessed through molecular docking. This study identified 395 methylation loci, 29 genes, and 5 proteins linked to disease using blood-derived QTL data. Replication confirmed 62 methylation loci, 5 genes, and 1 protein were associated with DN. Seven methylation loci influenced four DN-related genes (*BICC1*, *ANKIB1*, *KIF11*, *GNAI2*). Kidney tissues from DN patients showed elevated *GNAI2* and *KIF11* but reduced *PEBP1*. IRF2 was identified as a potential key transcription regulator. Drug predictions suggested calycosin, nitrofural, and nobiletin could target PEBP1, GNAI2, and KIF11, respectively. This SMR study suggested the causal role of circadian rhythm dysfunction in DN. Future research should focus on identifying specific genetic drivers, and exploring therapeutic strategies targeting circadian pathways to mitigate DN progression.

## Introduction

Diabetic nephropathy (DN), a microvascular complication of diabetes, is a leading global cause of end-stage renal disease (ESRD) [1], affecting 30%-40% of diabetic patients and accounting for about one-third of chronic kidney disease (CKD) cases [2]. Despite its high prevalence, current diagnostic markers, such as albuminuria, estimated glomerular filtration rate (eGFR), lack sensitivity [3]. On the therapeutic front, current treatments primarily target hyperglycemia, hypertension, and dyslipidemia but fail to address specific molecular mechanisms underlying DN progression [4]. Consequently, there is an urgent need not only for DN-specific biomarkers and mechanism-based therapeutics, but also for a deeper understanding of how these genes influence key clinical manifestations and systemic metabolic regulation. Such integrative insight will bolster both early detection strategies and the development of precision interventions.

Recent studies have investigated the connection between DN and circadian rhythm genes [5]. The circadian rhythm is an endogenous oscillator that coordinates diverse biological processes, ensuring synchronization between internal physiology and the external environment. It plays a crucial role in regulating metabolic processes, including glucose and lipid metabolism, and its disruption has been linked to metabolic disorders such as type 2 diabetes, obesity, and cardiovascular diseases [6]. Circadian rhythm disruptions may also contribute to DN through pathways involving metabolic dysregulation, inflammation, and oxidative stress. Studies have highlighted the role of circadian rhythms in maintaining normal renal function and suggested potential links to DN, emphasizing the need for further clinical and mechanistic exploration [7]. These approaches are susceptible to confounding and reverse causality, making it challenging to determine if circadian pathway alterations causally drive DN or are merely a symptom. Identifying the specific molecular players with a causal link is essential for developing targeted interventions.

To overcome the limitations of observational studies, such as confounding and reverse causality, we employed summary-data-based Mendelian Randomization (SMR) [8]. SMR integrates genome-wide association study (GWAS) summary statistics with molecular quantitative trait loci (QTL) data to infer causal relationships, while the integrated heterogeneity in dependent instruments (HEIDI) test helps distinguish these signals from linkage or pleiotropic effects [9]. To date, no comprehensive SMR analysis has systematically investigated the causal role of circadian rhythm-related genes in the development of diabetic nephropathy. Therefore, this study aimed to systematically identify circadian rhythm genes associated with DN risk through multi-omics data integration. Our findings offer a more comprehensive framework for understanding and intervening in DN progression.

## Materials and methods

### Study design

**Figure 1.**
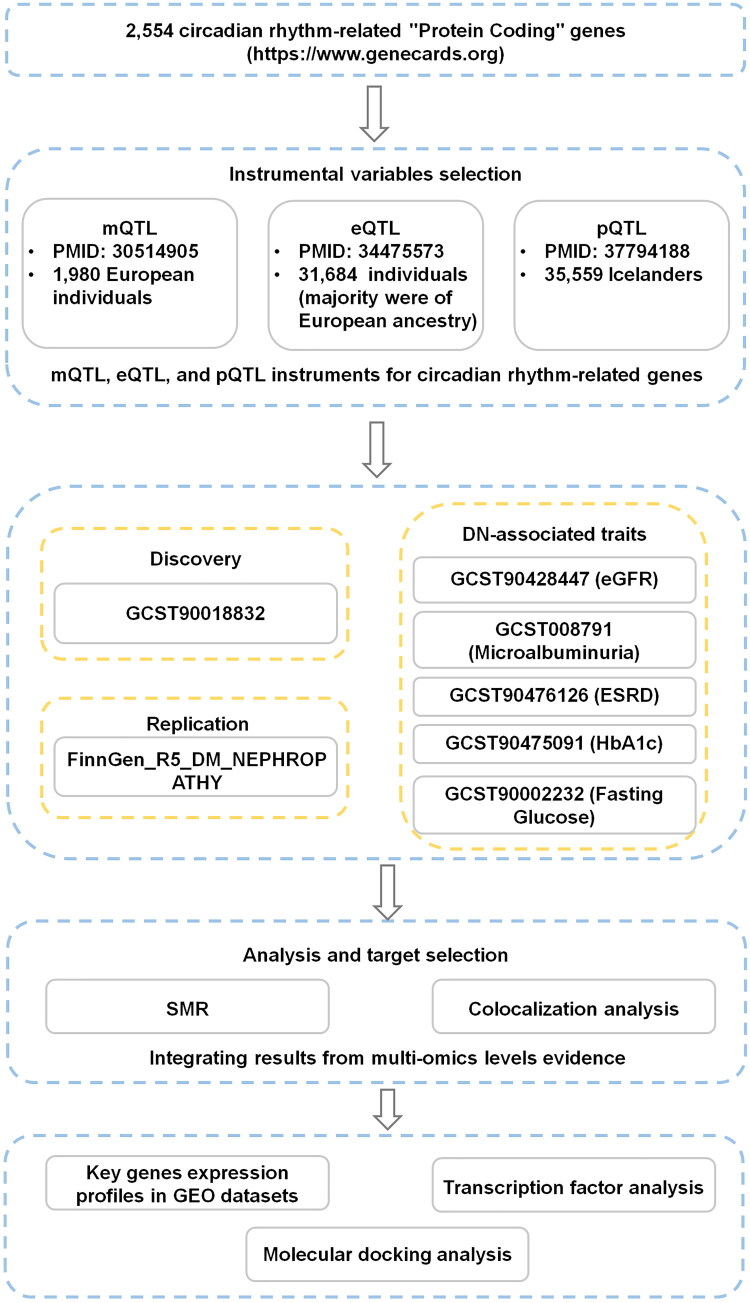
Flowchart of the analyses performed.

Figure 1 illustrates the study design. We employed SMR and colocalization analyses to investigate causal relationships between genetically predicted circadian rhythm-related molecular traits (methylation quantitative trait loci [mQTLs], expression quantitative trait loci [eQTLs], and protein quantitative trait loci [pQTLs]) and DN risk. GWAS summary statistics for DN were sourced from the GWAS Catalog (discovery) and FinnGen (replication). We first performed SMR to identify associations, followed by colocalization to distinguish causality from linkage disequilibrium. Expression of key genes was validated in kidney tissue samples from the Gene Expression Omnibus (GEO). Finally, we predicted potential drugs for key targets and evaluated their interactions *via* molecular docking. The study adheres to STROBE-MR guidelines.

### Data sources and selection of circadian rhythm-related genes

To identify circadian rhythm-related genes, we performed a systematic search in the GeneCards database using the keywords ‘circadian rhythm’ and ‘circadian clock,’ restricted to protein-coding genes. Gene Ontology (GO) and Kyoto Encyclopedia of Genes and Genomes (KEGG) enrichment analyses were subsequently conducted using the “clusterProfiler” R package to evaluate the biological relevance of the identified genes to molecular clock regulation. To further assess the reliability of this initial pool, we benchmarked our list against five expert-curated circadian pathways obtained from the Molecular Signatures Database (MSigDB) using the “msigdbr” R package. These pathways included BIOCARTA_CIRCADIAN_PATHWAY, WP_CIRCADIAN_RHYTHM_GENES, KEGG_CIRCADIAN_RHYTHM_MAMMAL, WP_EXERCISEINDUCED_CIRCADIAN_REGULATION, and PID_CIRCADIAN_PATHWAY.

### Data sources for DNA methylation, gene expression and protein quantitative trait loci

Publicly available summary statistics from studies with prior ethical approval were used (Table S1). Blood mQTL data were from McRae et al. [10], blood eQTL data from the eQTLGen consortium (*n* = 31,684) [11], and circulating pQTL data from a study in Iceland (*n* = 35,559) [12]. These datasets were primarily derived from populations of European ancestry.

DN GWAS data for discovery were sourced from the GWAS Catalog (GCST90018832, 1,032 cases/451,248 controls) (https://www.ebi.ac.uk/gwas/) [13], with replication from FinnGen R5 DM_NEPHROPATHY (3,283 cases/210,463 controls) (https://www.finngen.fi/en). Across these large GWAS datasets, DN was defined using clinically derived phenotyping strategies that combined diabetes status with indicators of kidney disease based on electronic health records or registry data. In the discovery dataset (GCST90018832), DN were identified using a high-fidelity deep-phenotyping approach that integrated multiple data sources, including past medical history, medication records, and text-mining from electronic medical records (EMR). This process involved mapping clinical diagnoses to the International Classification of Diseases (ICD-10) and Phecode systems to ensure diagnostic precision across different biobanks. By employing the hierarchical grouping of Phecodes, the study systematically aggregated related clinical conditions while defining stringent control groups to enhance the precision of genetic associations [13]. In FinnGen R5, diabetic nephropathy (DM_NEPHROPATHY) was defined using registry-based clinical diagnoses of diabetic kidney disease. The endpoint integrates related severe renal outcomes, including diabetic kidney failure (DM_KIDNEYFAIL), dialysis (DM_DIALYSIS), and kidney transplantation (DM_KIDNEYTRANSPLANT), to capture advanced manifestations of diabetic renal complications. To explore effects on renal function, we also analyzed DN-related outcomes including eGFR [14], microalbuminuria [15], ESRD [16], HbA1c levels [16] and fasting glucose levels [17].

### SMR analysis

To infer causal relationships between molecular traits and DN, we employed SMR, a method that uses aggregated GWAS findings and is well-suited for multi-omics analysis. We utilized mQTL, eQTL, and pQTL data as exposures and DN as the outcome, performing the analysis with SMR software (v1.3.1).

To ensure the validity of causal inference, our SMR analysis strictly adhered to the three fundamental MR assumptions, evaluated as follows: (1) Relevance assumption: We selected the most significant cis-QTL (Top SNP) within *a* ± 1000 kb window of each target gene as the instrumental variable (IV), requiring a genome-wide significance threshold of *p* < 5.0 × 10^−8^. (2) Independence assumption: By restricting IVs to cis-acting variants, we reduced the risk of confounding by distant trans-regulatory effects or complex systemic factors, thereby supporting the independence of the instruments from potential confounders. (3) Exclusion restriction assumption: To validate the exclusion restriction assumption, we employed the HEIDI (Heterogeneity in Dependent Instruments) test to safeguard our causal inference against horizontal pleiotropy and confounding by linkage disequilibrium (LD). Since SMR analysis typically utilizes a single top-associated SNP as the instrumental variable, traditional pleiotropy assessments like MR-Egger are not applicable. Instead, the HEIDI test ensures the robustness by evaluating the consistency of effect size patterns across all SNPs within the local LD region. Specifically, if the association between the lead SNP and the outcome is truly mediated by the target gene, the association signals of other SNPs in the same LD block should remain proportional to that of the lead SNP. Any significant deviation indicates that the observed association is likely driven by horizontal pleiotropy, where the variant affects the outcome *via* alternative biological pathways, or by distinct causal variants in LD, where the eQTL and GWAS signals are independent but physically close. In the case of LD, this is characterized in the Locus Plot by a physical displacement between the eQTL and GWAS signals. Even if both signals are significant, their peaks (the most significant SNPs) do not align at the same genomic coordinate. This indicates that while the two traits are statistically correlated due to their proximity on the chromosome, they are likely driven by two different underlying causal variants. In the case of horizontal pleiotropy, the lead SNP for both eQTL and GWAS might be the same (the peaks align), but the surrounding SNPs do not follow a consistent ratio. When plotting the effect sizes (beta_eQTL vs. beta_GWAS), the points appear scattered rather than forming a clean diagonal line. This suggests the lead SNP is working two jobs affecting the gene expression through one path and the disease through a completely separate, independent biological pathway. Thus, only loci with P_HEIDI > 0.01 were retained as supporting the exclusion restriction [10]. Finally, all SMR P-values were adjusted for multiple testing using the false discovery rate (FDR) method.

### Colocalization analysis

To assess potential genetic co-localization between two related phenotypes, colocalization analysis can be employed to determine whether they are influenced by common genetic causal variants within a specific genomic region. We explored five exclusivity hypotheses in the colocalization analyses: 1) no SNP association with either trait (H0); 2) association with the first trait solely (H1); 3) association with the second trait solely (H2); 4) distinct associations with both traits *via* separate variants (H3); 5) a common variant linking both traits (H4). The window sizes for defining the colocalization regions were set at ± 1000 kb for pQTL-GWAS, ± 1000 kb for eQTL-GWAS, and ± 500 kb for mQTL-GWAS analyses. We adopted a posterior probability threshold of PPH4 > 0.5 to identify suggestive evidence of shared causal variants. This threshold was chosen within our multi-omics framework to balance discovery power and specificity, particularly considering the varying sample sizes across mQTL, eQTL, and pQTL datasets.

### Expression profiles of key genes and transcription factor prediction

The expression of key genes (*BICC1, ANKIB1, KIF11, PEBP1*, and *GNAI2*) was validated using the publicly available GEO dataset GSE96804 [18]. This dataset contained glomeruli samples from controls and patients with biopsy-confirmed DN, staged as early (eGFR > 90 mL/min) or late (eGFR 15–60 mL/min) [18]. Differential expression was analyzed using the “limma” package (Wilcoxon *p* < 0.05) and visualized with “ggplot2” package.

To identify potential upstream regulators of the prioritized genes, we utilized the Subnetwork Analysis module of the KnockTF 2.0 database (http://www.licpathway.net:8081/KnockTFv2/). Each candidate gene was queried individually using default parameters (Homo sapiens, top 100 edges) to construct regulatory subnetworks based on integrated perturbation datasets, including TF knockdown/knockout RNA-seq and ChIP-seq data. Regulators were prioritized according to network topology metrics, including Degree and Betweenness, as well as their occurrence across multiple target genes. To enhance biological relevance, only transcription factors associated with both circadian rhythm and diabetic nephropathy were retained for further analysis.

### Drug-protein interaction

To explore potential therapeutic relevance, putative drug candidates targeting each key gene were predicted using the Enrichr platform (DSigDB database), and the top five candidates were selected based on P-values [19]. 3D structures for drugs (ligands) and proteins were obtained from PubChem and the Protein Data Bank (PDB), respectively. The structures were prepared for docking by converting ligands with Open Babel 2.4.1 and preprocessing proteins (e.g., removing water, adding hydrogens) in PyMOL 2.6.0. Molecular docking was then performed using AutoDock Vina. Binding affinity was used to evaluate the interactions. According to commonly adopted criteria, binding energies < −5.0 kcal/mol are generally considered indicative of good binding activity, whereas values < −7.0 kcal/mol suggest strong binding [20].

### Statistical analysis

All statistical analyses were performed in R (v4.3.0). Manhattan and forest plots were generated using the “CMplot” and “forestplot” packages [21].

## Ethics approval and consent to participate

This study utilized publicly available, aggregated data from studies that had already received ethics approval; therefore, no additional institutional review was required.

## Results

### Circadian rhythm-related gene expression and DN

**Figure 2.**
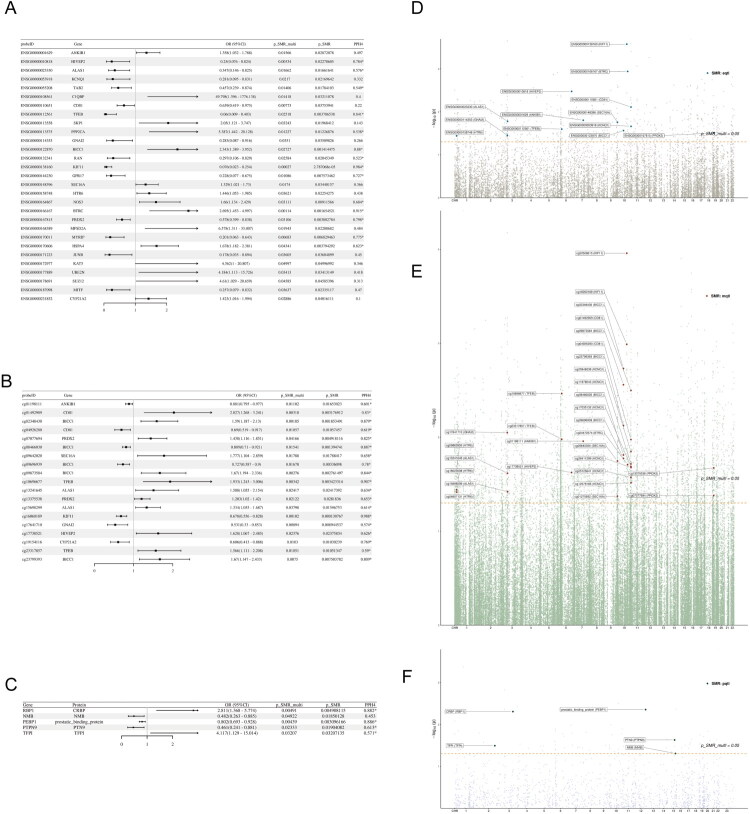
Causal associations between circadian rhythm-related gene expression, methylation, protein abundance and DN. (A) Forest plot of SMR analysis results between representative circadian rhythm-related gene expression and DN. (B) Forest plot of SMR analysis results between representative circadian rhythm-related DNA methylation and DN. (C) Forest plot of SMR analysis results between representative circadian rhythm-related protein abundance and DN. (D) Manhattan plot showing the distribution of eQTLs on chromosomes. (E) Manhattan plot showing the distribution of mQTLs on chromosomes. (F) Manhattan plot showing the distribution of pQTLs on chromosomes.

A total of 29 genes associated with DN were identified, with 15 genes showing a positive association with an increased risk of DN (P-SMR-multi < 0.05, P-SMR < 0.05, and P-HEIDI > 0.01) (Table S2-S3). Furthermore, the colocalization analysis indicated that 4 genes (*BICC1*, *GPR17*, *PRDX2* and *JUNB*) had loci associated with DN within the colocalization region of the corresponding SNPs (PPH4 > 0.5) (Figure 2A). In the FinnGen_dataset, 5 genes (*ANKIB1*, *BICC1*, *CYP21A2*, *GNAI2*, *KIF11*) were validated as being associated with DN risk (Table S4-S5). By integrating multi-omics causal evidence with clinical phenotypic data, these five key genes demonstrated robust associations with DN risk and renal functional status (Table 1). A distribution of eQTLs across chromosomes was shown using a Manhattan plot in Figure 2D.

**Table 1.** The summary information of the five key signals associated with DN.

Gene (Key CpG site)	SMR analysis with DN (Discovery Level)	Colocalization (Discovery)	SMR analysis with DN (Replication Level)	SMR analysis with eGFR	SMR analysis with microalbuminuria	SMR analysis with ESRD	SMR analysis with HbA1c	SMR analysis with fasting glucose
BICC1 (cg09696939)	√ (mQTL, eQTL)	√ (mQTL, eQTL)	√ (mQTL, eQTL)	+	+	+	/	/
ANKIB1 (cg01198111)	√ (mQTL, eQTL)	√ (mQTL)	√ (mQTL, eQTL)	+	/	–	/	/
KIF11 (cg16O6O189)	√ (mQTL, eQTL)	√ (mQTL, eQTL)	√ (mQTL, eQTL)	/	/	–	/	–
PEBP1	√ (pQTL)	√ (pQTL)	√ (pQTL)	/	/	/	/	/
GNAI2 (cg17641710)	√ (mQTL, eQTL)	√ (mQTL)	√ (mQTL)	/	/	/	/	/

### Circadian rhythm-related gene methylation and DN

We identified a total of 395 methylation sites corresponding to 199 genes associated with DN. A subset of significant mQTL associations is presented in Figure 2B for clarity, with the complete results provided in Table S6. Among these features, 248 sites corresponding to 127 genes were found to have strong evidence supporting colocalization (PPH4 > 0.5) (Figure S1). Notably, 14 DN-associated genes (*ANKIB1*, *BICC1*, *BTRC*, *CYP21A2*, *HTR6*, *SEC16A*, *ALAS1*, *CD81*, *GNAI2*, *HIVEP2*, *KCNQ1*, *KIF11*, *PRDX2* and *TFEB*) were uncovered across both mQTL and eQTL levels. A distribution of mQTLs across chromosomes was shown using a Manhattan plot in Figure 2E. In the FinnGen_dataset, 62 methylation sites corresponding to 40 genes were validated, such as *ANKIB1*, *BICC1*, and *KIF11* (Table S7-S8).

### Circadian rhythm-related protein abundance and DN

The results of the SMR analysis, including the causal relationship between circadian rhythm-related protein abundance and DN can be further understood by referring to Figure 2C. Utilizing same criteria, 5 proteins linked to genes associated with DN were successfully identified. Notably, among these proteins, 4 proteins (PEBP1, PTPN9, RBP1, TFPI) demonstrated compelling colocalization evidence (PPH4 > 0.5) (Table S9-S10). The validation of PEBP1 was successfully demonstrated in the FinnGen dataset (Table S11-S12). A distribution of pQTLs across chromosomes was shown using a Manhattan plot in Figure 2F.

### Integrating evidence from multi-omics levels

We further explored the regulation of blood methylation on the expression of key circadian rhythm genes in DN through SMR analysis by integrating mQTL and eQTL data. We identified 29 methylation sites corresponding to 13 genes that potentially have significant implications in DN (Table S13). Nevertheless, no significant associations were observed when combining eQTLs and pQTLs data. Integration of SMR analysis results from blood mQTLs, eQTLs, and pQTLs revealed four intersecting genes (*ANKIB1*, *BICC1*, *GNAI2*, *KIF11*) that may have causal relationship between their corresponding methylation sites and gene expression. Moreover, the associations between these four genes and DN were replicated in the FinnGen dataset, with *BICC1* exhibiting additional colocalization evidence with DN risk. For further insight into the SMR analysis results of *KIF11*, *ANKIB1*, *BICC1*, and *GNAI2* in eQTL and mQTL, supplementary information is available in Figures S2–S5.

### Causal associations between key signals and DN-related phenotypes

**Figure 3.**
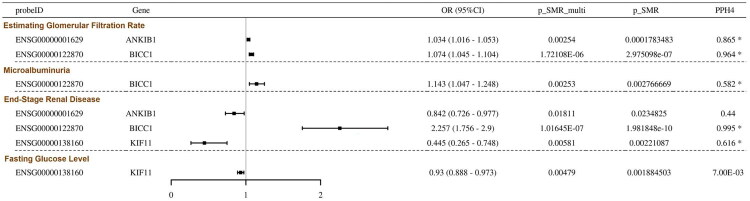
Causal associations between the expression of key signals and DN-related traits. A forest plot of SMR analysis results between *ANKIB1*, *BICC1* and *KIF11* expression and eGFR, microalbuminuria, ESRD and fasting glucose levels.

To clarify whether the observed genetic associations reflect causal effects on DN-related traits, we performed SMR analyses regarding the expression of *BICC1*, *ANKIB1*, *KIF11*, *PEBP1* and *GNAI2* to clinically relevant phenotypes, such as eGFR, microalbuminuria, ESRD, HbA1c and fasting glucose. The results indicated that the expression of *BICC1* was positively associated with eGFR, microalbuminuria and ESRD (Figure 3; Table S14). Conversely, *ANKIB1* expression levels correlated positively with eGFR yet inversely with the risk of ESRD, whereas *KIF11* expression was negatively linked to ESRD development and fasting glucose concentrations (Figure 3; Table S14). Collectively, these causal relationships underscore that the expression of these genes was intimately coupled to renal functional status.

### Functional and therapeutic investigation of key genes

**Figure 4.**
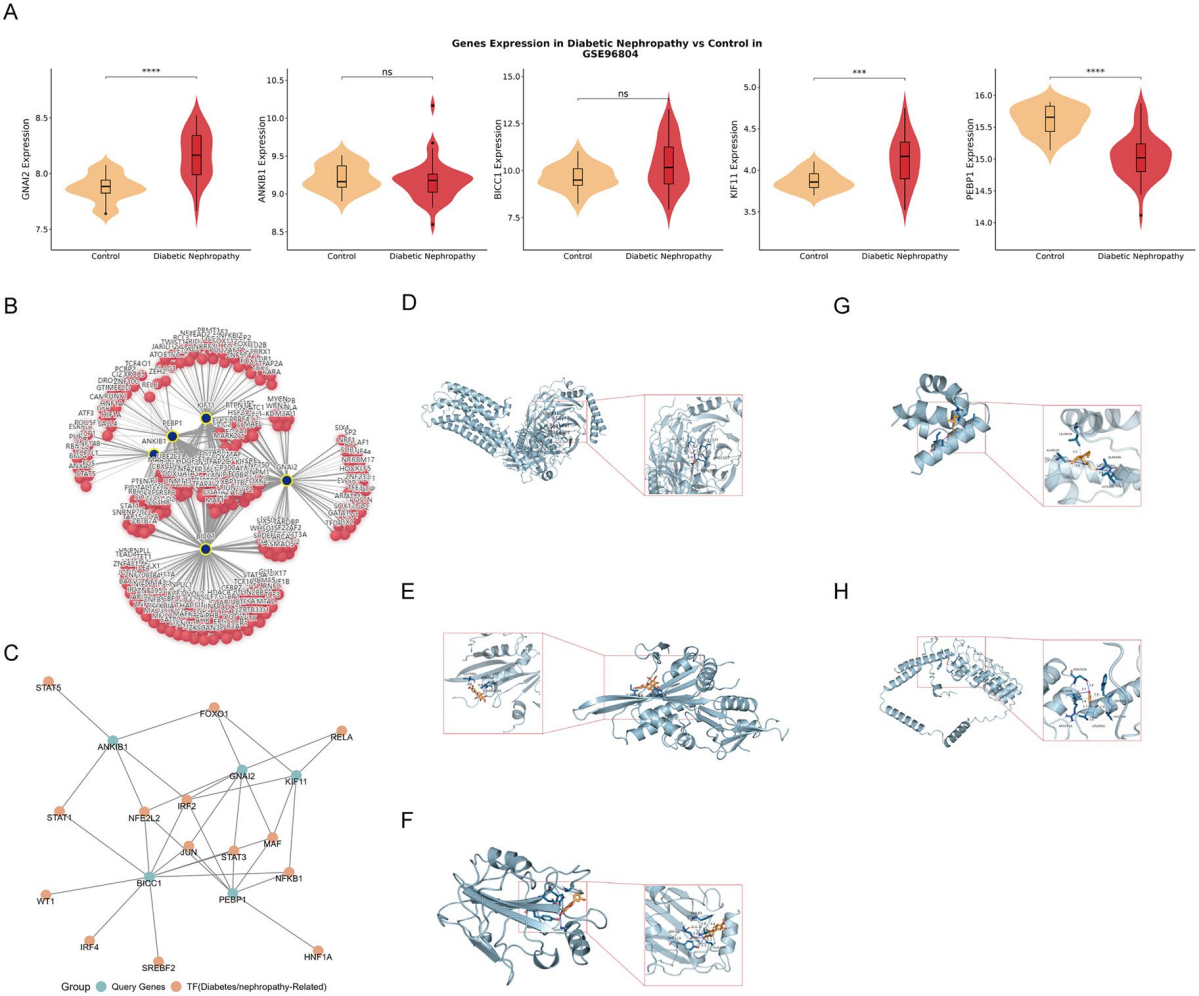
Validation of gene expression, transcriptional regulation, and molecular docking of key circadian rhythm-related genes. (A) Violin plots illustrating the differential expression of *GNAI2*, *ANKIB1*, *BICC1*, *KIF11*, and *PEBP1* in glomerular tissues from DN patients compared to controls (GSE96804). The central box represents the interquartile range. Statistical significance was determined using the Wilcoxon test (ns: non-significant; ***p < 0.001; ****p < 0.0001). (B) The global transcription factor (TF)-gene regulatory network. Red nodes represent potential TFs, and blue nodes represent the target circadian genes. Each line indicates a predicted regulatory relationship. (C) A filtered sub-network highlighting TFs specifically associated with both circadian rhythms and DN (e.g., IRF2), showing their connections to the identified target genes. (D–H) Visualization of the molecular docking modes between the five key targets and their top predicted small-molecule drugs. The 3D structures display the binding interactions for: (D) GNAI2 with nitrofural; (E) KIF11 with nobiletin; (F) PEBP1 with calycosin; (G) BICC1 with cyclosporin A; and (H) ANKIB1 with acetaminophen.

To investigate the biological and therapeutic relevance of the five key genes implicated in DN pathogenesis (*BICC1, ANKIB1, KIF11, PEBP1*, and *GNAI2*), we conducted a series of in silico analyses. First, expression validation in renal tissues (GEO: GSE96804) confirmed their dysregulation in DN, revealing significantly elevated *GNAI2* and *KIF11* and reduced *PEBP1* expression in patient glomeruli (Figure 4A).

Next, transcription factor (TF) prediction identified IRF2 as a potential key regulator of these genes (Figure 4B-4C). To validate these findings within a disease-specific context, we analyzed the expression correlation between IRF2 and its predicted targets in human DN samples from the GSE96804 dataset. Pearson correlation analysis revealed a significant positive association between *IRF2* and *GNAI2* (*r* = 0.485, *p* = 0.0013, Figure S6A). Additionally, *BICC1* and *PEBP1* showed marginal correlation trends (*p* = 0.059 and *p* = 0.073, respectively, Figure S6B-S6C). In contrast, no statistically significant correlations were observed for *KIF11* (Figure S6D) and *ANKIB1* (Figure S6E). These results support the biological relevance of the IRF2-mediated regulatory network, particularly the IRF2-GNAI2 axis, in the progression of DN. However, given the complexity of clinical samples and potential compensatory mechanisms, the direct binding of IRF2 to all five promoters remains to be experimentally confirmed.

Finally, molecular docking predicted strong binding affinities for several small molecules, including calycosin with PEBP1 (−9.129 kcal/mol) and nobiletin with *KIF11* (−7.205 kcal/mol), which indicate strong binding, as well as nitrofural with *GNAI2* (−6.958 kcal/mol), consistent with good binding activity, suggesting they may represent potential therapeutic targets (Figure 4D-4H; Table S15).

### Systematic validation and biological plausibility of circadian-related candidates

We initially identified 2,554 high-confidence protein-coding genes associated with circadian rhythms *via* GeneCards to ensure a comprehensive candidate pool (Table S16). To validate the biological plausibility of this selection, GO and KEGG enrichment analyses were performed. The initial set showed extensive enrichment in molecular clock modulation, photoperiodic entrainment, and the sleep-wake cycle (Table S17-S18), while the final SMR-prioritized subset maintained strong functional consistency, particularly within GO biological process terms (Table S19-S20). Furthermore, cross-validation against the MSigDB gold standard (Table S21) demonstrated that our initial screening captured 97.14% (238/245) of established circadian regulators (Figure S7A), with 21 SMR-prioritized genes directly overlapping with these expert-curated pathways (Figure S7B). Collectively, these findings establish a robust, closed-loop validation framework, confirming that our prioritized candidates are biologically credible components of the circadian system and supporting a potential causal link to DN.

## Discussion

In this study, SMR and colocalization analyses identified several circadian rhythm-related genes (*ANKIB1*, *BICC1*, *GNAI2*, *KIF11* and *PEBP1*) showing significant association with the risk of DN. These findings establish a genetic framework linking circadian disruption to DN, offering potential targets for therapeutic intervention.

BICC1, an evolutionarily conserved RNA-binding protein, is crucial for various biological processes, including kidney development and the regulation of circadian rhythms [22]. Functionally, *BICC1* regulates mRNA stability and translation, modulating signaling pathways in epithelial polarity and nephron morphogenesis [23]. Experimental studies, such as *BICC1* knockout in mice leading to diabetes phenotypes [24], and human genetic studies linking *BICC1* variants to CKD outcomes [25], support its potential involvement in renal and metabolic dysfunction. In this study, *BICC1* was causally associated and colocalized with DN risk across mQTL and eQTL levels, further supported by replication studies. This suggested that *BICC1* might contribute to the occurrence of DN. Notably, *BICC1* expression showed positive associations with both eGFR and DN-related traits (ESRD and microalbuminuria). This seemingly paradoxical pattern may reflect the compensatory hyperfiltration phase often observed in early-stage chronic kidney disease, where elevated GFR initially manifests as increased filtration due to glomerular hypertension, however, this sustained glomerular hypertension ultimately drives progression to proteinuria and renal failure [26]. Despite the causal associations between *BICC1* expression and DN-related traits, its expression was comparable between kidney tissues from both controls and DN patients. This controversy may stem from the fact that blood eQTL signals often diverge from those observed in kidney tissue, while sample-size constraints and inter-study heterogeneity further dilute statistical power. Moreover, *BICC1* may exert stage- or cell-type-specific effects within the diabetic kidney. Notwithstanding the lack of direct tissue validation, convergent MR and colocalization evidence across multiple levels suggest its relevance in DN pathogenesis.

*ANKIB1* (ankyrin repeat and IBR domain containing 1) is an E3 ubiquitin ligase known for its roles in inflammation and the regulation of circadian rhythms [27]. By degrading or modifying clock-associated molecules, it may modulate circadian-controlled metabolic and inflammatory responses [27]. While prior evidence specifically linking *ANKIB1* to DN is limited, our multi-omics analysis revealed a causal association. Specifically, genetically predicted hypomethylation at the cg01198111 locus was associated with increased *ANKIB1* expression, and this genetically-driven increased expression was causally linked to an elevated risk of DN. Moreover, the expression of *ANKIB1* was associated with higher eGFR and reduced risk for ESRD, implying its potential protective effect. Therefore, ANKIB1 may play a multifaceted role in DN, in that its influence may be stage-dependent, shifting from renoprotective to neutral or even adverse as disease progresses. On the other hand, the blood-based eQTL signal may reflect a systemic compensatory up-regulation rather than true renal expression. Thus, future studies may focus on the dynamic role of ANKIB1 in DN progression and provide novel genetic support for its involvement.

We found a causal link between higher PEBP1 protein levels and reduced DN risk. PEBP1 is a multifunctional regulatory protein known to inhibit the Raf-1/MEK/ERK pathway and play roles in metabolic and inflammatory processes [28]. PEBP1 also modulates circadian regulation through kinase signaling pathways that intersect with core clock components [29]. By influencing MAPK-mediated synchronization of peripheral clocks with metabolic cues, PEBP1 may regulate circadian-controlled metabolic responses in renal cells under hyperglycemic conditions. Emerging evidence highlights its significance in metabolic and inflammatory pathways, particularly in diabetic nephropathy. For example, *PEBP1* expression was significantly downregulated in renal tissues in experimental DN models, correlating with disease severity [30]. The downregulation of PEBP1 could contribute to chronic inflammation in renal tissues, as its downregulation could be accompanied by increased NF-κB activity and elevated urinary protein excretion and glomerulosclerosis [30]. Mechanistically, PEBP1 could contribute to the maintenance of mitochondrial function by enhancing eIF2α phosphorylation under hyperglycemia, which promotes selective translation of stress-responsive genes like ATF4 [31]. In PEBP1-deficient renal cells, this adaptive mechanism is impaired, leading to accelerated cell death [31]. Our findings showed the levels of PEBP1 were negatively associated with DN risk. Furthermore, decreased PEBP1 expression in kidney tissues from human DN patients supports its potential protective role and therapeutic relevance. Future research should investigate the specific protective mechanisms of PEBP1 in DN and explore its potential as a therapeutic target.

For *KIF11* and *GNAI2*, our findings revealed a key discrepancy between systemic and local tissue effects. KIF11 is a microtubule motor protein essential for mitotic spindle formation and cell cycle progression [32]. While previous studies link *KIF11* to increased type 2 diabetes risk and more severe DN progression, our genetic analysis revealed a protective association [33]. Previous research has revealed that increased expression of the *KIF11* gene was associated with more severe progression of DN [34,35]. By contrast, our findings revealed that *KIF11* was negatively associated with DN at m/eQTLs across both discovery and replication dataset, with its expression negatively associated with ESRD risk, fasting glucose and Hb1Ac levels. However, its expression was elevated in kidney tissues from DN patients. This discrepancy between blood eQTL data and transcriptome could be attributed to the tissue-specific expression and role of *KIF11*. Similarly, *GNAI2* encodes a Gαi subunit involved in GPCR-mediated signaling, which is implicated in other diabetic complications like retinopathy and atherosclerosis[36,37]. Previous experimental research has shown that elevated expression of *GNAI2* is associated with increased apoptosis of retinal ganglion cells and exacerbated development of atherosclerosis [38]. Despite a lack of direct evidence in DN, our analysis identified a genetically predicted protective role for *GNAI2* in blood against DN risk. Yet again, this contrasted with its increased expression in DN kidney tissue. We propose that this tissue-specific discordance does not invalidate the causal interpretation but rather reflects fundamentally different biological dimensions through two non-exclusive mechanisms. First, the elevated expression in DN tissues likely represents a reactive compensatory up-regulation rather than a primary pathogenic driver. While our SMR evidence suggests that higher lifelong baseline expression of these genes exerts a protective effect against DN onset, their increased levels in diseased tissues may signify the kidney’s adaptive response to hyperglycemia-induced stress. Given that *KIF11* has been implicated in regulating cell cycle progression [32], its up-regulation in DN tissues may represent a compensatory response aimed at promoting cell-cycle entry and proliferation to support the repair of injured tubular epithelial cells or podocytes. Similarly, given that *GNAI2* has been implicated in inflammatory responses to hyperglycemic stress [38], its up-regulation in DN tissues may reflect a compensatory response induced by oxidative stress and inflammatory cytokines, potentially activating anti-inflammatory feedback mechanisms to maintain podocyte integrity. In this context, the blood-based genetic signal may capture systemic protective capacity against the initial injury, whereas increased local tissue expression may reflect a failed or exhausted compensatory response representing a late-stage reparative attempt within the pathological microenvironment. Second, this inconsistency may reflect a temporal hierarchy in causal inference. Genetic evidence provides insight into causal precedence based on lifelong baseline expression, whereas tissue transcriptomics captures a synchronized snapshot of an established disease state and therefore mainly represents associative patterns. Given the chronic nature of DN, the pathological milieu characterized by hyperosmolar stress and persistent inflammation can substantially remodel the gene expression landscape. Consequently, the divergence between SMR findings and observational transcriptomic results may highlight the dynamic and stage-specific roles of these genes as the disease progresses from initial susceptibility to chronic maladaptation. These hypotheses, while biologically plausible based on the known roles of *KIF11* in cell cycle and *GNAI2* in stress response, remain speculative and warrant further longitudinal single-cell profiling and lineage-tracing studies to fully dissect their functional transitions across disease progression. Nevertheless, the robust genetic links of *KIF11* and *GNAI2* to DN and its related metabolic traits establish them as pivotal nodes in pathogenesis, warranting focused mechanistic investigation into their context-dependent roles.

Based on our integrative network analyses, we next uncovered *IRF2* as the potential key transcriptional factor regulating the expression of *BICC1*, *ANKIB1, KIF11, PEBP1* and *GNAI2.* To bridge the gap between database-driven predictions and disease-specific biological relevance, we further validated these regulatory links using clinical transcriptomic data from the GSE96804 dataset. Our correlation analysis revealed a robust positive association between *IRF2* and *GNAI2* in DN patients. The high significance of this correlation in human renal tissues provides strong evidence that IRF2 may indeed act as a proximal regulator of *GNAI2* under diabetic conditions. Additionally, the marginal correlation trends observed for *BICC1* and *PEBP1* further suggest a potential, albeit perhaps more complex, regulatory influence. Although the correlations for *KIF11* and *ANKIB1* did not reach statistical significance, potentially due to the inherent noise in clinical samples or cell-type-specific regulatory dynamics, the overall concordance, particularly the strength of the IRF2-GNAI2 axis, underscores the validity of our TF-target predictions. Given its established roles in inflammation and proliferation, IRF2 appears to act as a crucial regulatory node that translates upstream circadian disruption into downstream transcriptional reprogramming that collectively drives DN progression.

In summary, these prioritized genes potentially represent a diverse functional spectrum linking circadian rhythms to DN pathogenesis. It is hypothesized that BICC1 and ANKIB1 may modulate the speed and amplitude of the circadian clock through post-transcriptional and post-translational modifications, thereby potentially influencing renal epithelial polarity and inflammatory homeostasis in a stage- or context-dependent manner. PEBP1 likely acts as a metabolic-circadian integrator that could protect the kidney by stabilizing mitochondrial function, regulating MAPK-mediated peripheral clock synchronization, and suppressing NF-κB-mediated inflammation under hyperglycemic stress. In contrast, KIF11 and GNAI2 appear to govern the transition between initial injury protection and late-stage compensatory repair, suggesting their involvement in cell-cycle progression and G-protein signaling. Collectively, these genes, potentially coordinated by the key transcription factor IRF2, may form a regulatory network that translates systemic circadian desynchrony into localized renal pathological processes.

Moreover, molecular docking analysis nominated candidate small-molecule binders of the key proteins, including calycosin, nitrofural and nobiletin. Notably, nobiletin has been known as a circadian modulator, underscoring its therapeutic potential and reinforcing the rationale for circadian-related genes in DN [39]. Detailed inspection of the binding pose indicates that nitrofural is deeply anchored within the hydrophobic pocket of GNAI2. The stability of this complex is reinforced by a network of specific interactions, most notably the hydrogen bonds and hydrophobic contacts with key residues (ARG150, SER189, VAL276 and ILE232). The involvement of these conserved residues suggests that nitrofural may interfere with the conformational transitions of GNAI2 required for its coupling with downstream effectors. Previous studies have highlighted that the S-nitrosylation of GNAI2 at Cys66 is a critical driver of diabetes-accelerated atherosclerosis *via* the CXCR5/Hippo/YAP axis [38]. Our docking data provide a structural rationale for the potential therapeutic efficacy of nitrofural. By occupying the GNAI2 binding pocket, nitrofural might sterically hinder the pathological S-nitrosylation process or disrupt the aberrant SNO-GNAI2/CXCR5 coupling, thereby restoring Hippo pathway activity and alleviating endothelial inflammation.

A major strength of this study is the use of the multi-omics SMR framework, which leverages genetic variation to infer causal relationships, providing stronger evidence than traditional observational studies. Identifying circadian rhythm-related molecular traits with putatively causal links to DN risk offers a robust genetic basis for developing novel therapeutic strategies and personalized medicine approaches. We acknowledge that the definition of DN varies slightly across different GWAS consortia. While most cohorts rely on ICD coding and electronic health records, variations in diagnostic sensitivity, such as biopsy-confirmed cases versus those based on billing codes, may introduce phenotype heterogeneity. Nevertheless, the high consistency of our SMR results across the discovery and replication datasets indicates that the identified circadian genes represent core drivers of diabetic kidney injury regardless of specific clinical sub-definitions. This cross-dataset validation mitigates the risk that our findings are artifacts of a single phenotypic classification system.

However, several limitations should be considered when extending our conclusions to diverse populations and molecular contexts. Firstly, the genomic and phenotypic data utilized in this study were predominantly derived from cohorts of European ancestry. This choice was necessitated by the current availability of resources because European-ancestry datasets provide the largest sample sizes and the most comprehensive multi-omics coverage available to ensure maximal statistical power. Nevertheless, this reliance poses an inherent limitation to the global generalizability of our findings. Given the global prevalence of DN, the genetic architecture and environmental modifiers may vary significantly across different ethnic groups. Therefore, future studies incorporating large-scale omics data from non-European populations including those of East Asian and African as well as Latin American descent are essential to perform trans-ethnic validation and confirm the universal transferability of these molecular insights. Secondly, although multi-omics data were integrated, no significant overlap of associations was observed between the eQTL and pQTL levels. This phenomenon may be attributed to several factors. One possible explanation is the difference in statistical power, as currently available pQTL datasets generally have smaller sample sizes than large-scale eQTL resources, potentially leading to false negatives where biologically relevant protein-level associations fail to reach stringent significance thresholds. Another explanation is the inherently complex and non-linear relationship between transcripts and proteins. In the context of diseases, processes such as mRNA stability, translational regulation, and post-translational modifications may partially decouple mRNA expression from protein abundance. From a multi-omics perspective, our analysis aims to identify potential regulatory nodes across molecular layers, and genes identified at the eQTL level may still represent plausible candidates for further functional validation even if they have not yet reached statistical significance in current pQTL datasets. Overall, this discordance underscores the complexity of the regulatory landscape and highlights the need for larger proteomic cohorts in future studies. Thirdly, the discrepancies observed between blood-based QTL analysis and kidney tissue validation for genes such as *GNAI2* and *KIF11* underscore the need for further validation. While these observations are biologically plausible given the known roles of *KIF11* in cell cycle and *GNAI2* in stress responses, they remain speculative. Future studies will first employ longitudinal single-cell profiling and lineage-tracing approaches to dissect the functional transitions of these genes across disease progression, followed by experimental validation using perturbation in renal cell lines and diabetic mouse models to confirm their impact on kidney injury. Fourthly, the molecular docking analysis does not account for pharmacokinetic properties such as bioavailability, toxicity, or *in vivo* efficacy. Accordingly, the identified compounds should be considered preliminary lead candidates requiring further optimization and experimental validation, including biochemical assays such as surface plasmon resonance binding tests and IC_50_ measurements to confirm binding affinity and inhibitory activity. Finally, the choice of colocalization thresholds warrants consideration. While a PPH4 > 0.8 is often used for high-confidence causal inference, we utilized a more inclusive threshold of 0.5 to facilitate exploratory multi-omics integration. The primary advantage of this approach is that it avoids the loss of biologically relevant signals in pQTL and mQTL datasets, which inherently possess lower statistical power than transcriptomic resources. By combining this with the HEIDI test for pleiotropy and linkage disequilibrium filtering, we maintain a balanced discovery pipeline. However, we acknowledge that a threshold of 0.5 provides suggestive rather than definitive evidence of colocalization. Consequently, the candidates identified through this approach should be interpreted as prioritized targets requiring further experimental validation to confirm their functional roles in DN pathology.

While this study provides a robust genetic framework linking circadian regulators to diabetic nephropathy, several future directions are warranted to translate these findings into clinical utility. First, functional validation remains the immediate priority. We plan to perform CRISPR/Cas9 or siRNA mediated knockdown or overexpression of *BICC1*, *KIF11*, and *GNAI2* in high-glucose-induced podocytes and proximal tubule cells, as well as in db/db mice, to assess their effects on apoptosis, fibrosis markers, and core circadian clock components. Concurrently, we will integrate single-cell RNA sequencing and spatial transcriptomics in db/db mice to characterize the spatiotemporal expression patterns and *in vivo* functions of these genes in renal tissues. Second, mechanistic deepening will be pursued. Transcriptional regulatory validation will be conducted using ChIP-seq and luciferase reporter assays to confirm whether transcription factors such as IRF2 directly drive *GNAI2* and other candidate genes expression. We will also investigate whether core circadian components, including CLOCK and BMAL1, modulate these pathogenic mediators, aiming to delineate the full molecular chain linking circadian disruption, gene dysregulation, and renal injury. Third, cross-ethnic validation is essential, given that current datasets are predominantly of European ancestry. We aim to replicate these findings in large East Asian and African ancestry cohorts as multi-omics datasets become available, ensuring the generalizability of the identified genetic markers. Fourth, preclinical and pharmacological optimization will focus on candidate compounds such as nitrofural. Structure-activity relationship studies will be conducted to enhance renal specificity and minimize toxicity. Complementary biochemical assays, such as binding affinity measurements and inhibitory activity tests, together with *in vivo* efficacy evaluations, will help translate computational predictions into experimental validation. Finally, clinical translation will explore the potential of these genes as early diagnostic biomarkers by assessing their expression or methylation levels in blood samples, and will investigate the feasibility of chronotherapy by optimizing drug administration timing according to circadian gene expression patterns to achieve precision medicine in diabetic nephropathy.

In conclusion, our study provides compelling genetic evidence for the causal involvement of specific circadian rhythm-related molecular factors in DN pathogenesis. These findings enhance our understanding of the complex interplay between circadian disruption and DN and identify promising targets for future research aimed at developing effective interventions. Functional validation studies in relevant models are essential next steps to translate these findings into clinical applications.

## Supplementary Material

Supplemental Material

Supplemental Material

Supplemental Material

Supplemental Material

Supplemental Material

Supplemental Material

Supplemental Material

Supplemental Material

Supplemental Material

Supplemental Material

Supplemental Material

Supplemental Material

Supplemental Material

Supplemental Material

Supplemental Material

Supplemental Material

Supplemental Material

Supplemental Material

Supplemental Material

Supplemental Material

Supplemental Material

Supplemental Material

Supplemental Material

Supplemental Material

Supplemental Material

Supplemental Material

Supplemental Material

Supplemental Material

## Data Availability

The data that support the findings of this study are openly available in Zenodo at https://doi.org/10.5281/zenodo.19058904, reference number 19058904. These data were derived from the following resources available in the public domain: the FinnGen research project (https://www.finngen.fi/en), the GWAS Catalog (https://www.ebi.ac.uk/gwas/), and the IEU OpenGWAS database (https://gwas.mrcieu.ac.uk/).
