# Supplementary figures and images for: Potential circadian rhythm-related pathogenic genes in diabetic nephropathy: a multi-omics Mendelian randomization study
Source: Ren Fail. 2026 May 24;48(1):2663248. doi: 10.1080/0886022X.2026.2663248 (PMC13202663; doi:10.1080/0886022X.2026.2663248)

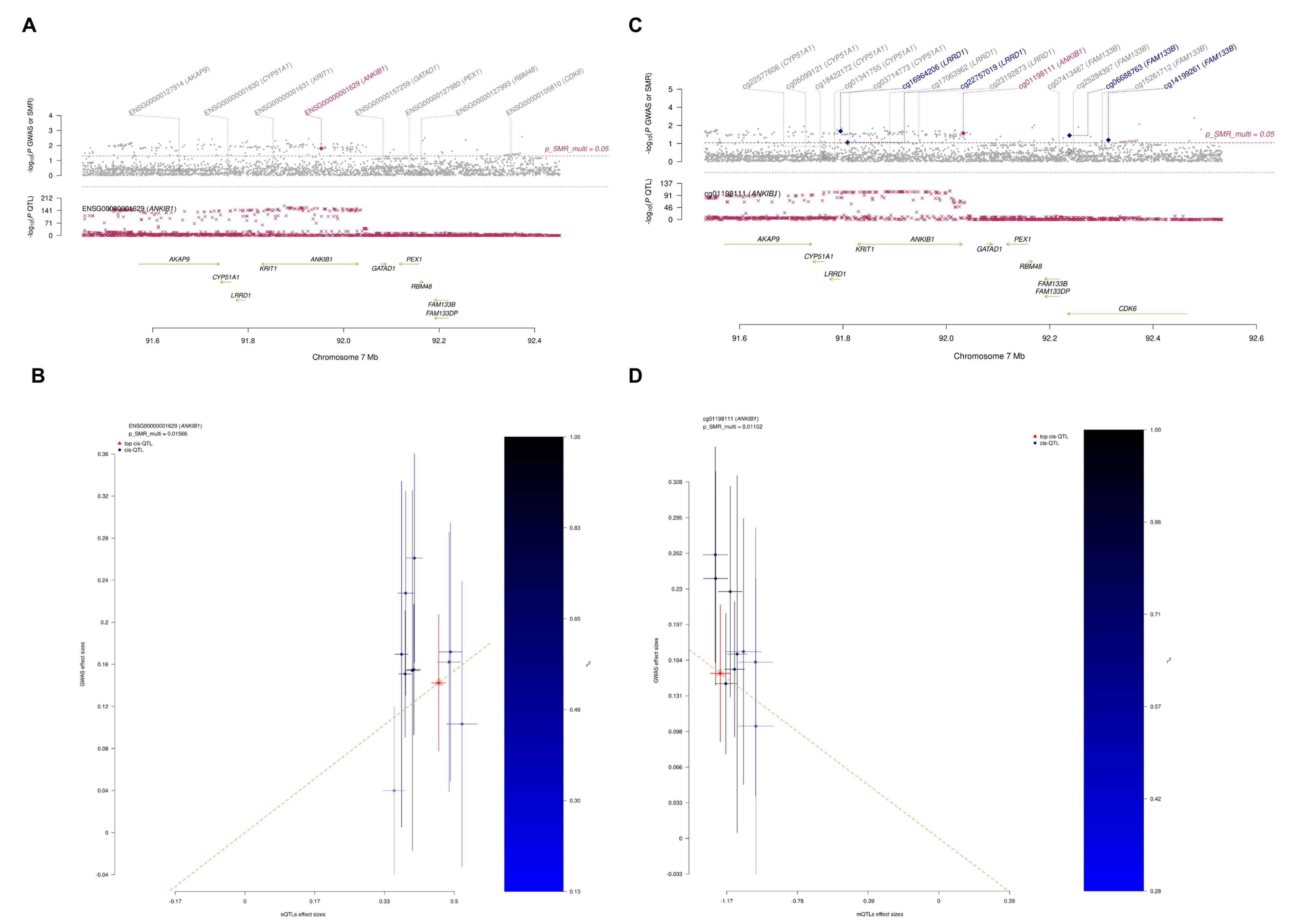

Supplement: Supplemental Material [file IRNF_A_2663248_SM8831.jpg]

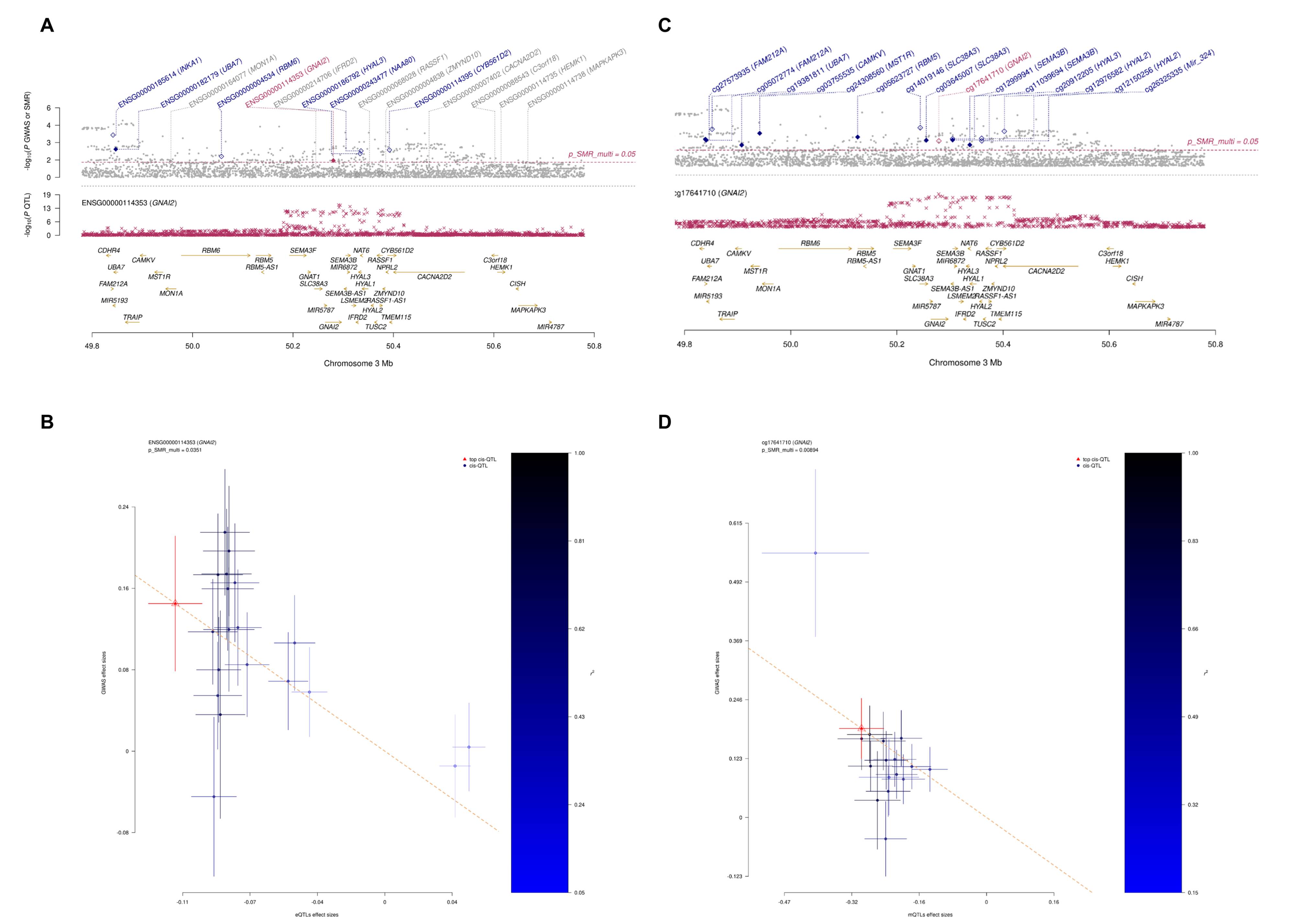

Supplement: Supplemental Material [file IRNF_A_2663248_SM8824.jpg]

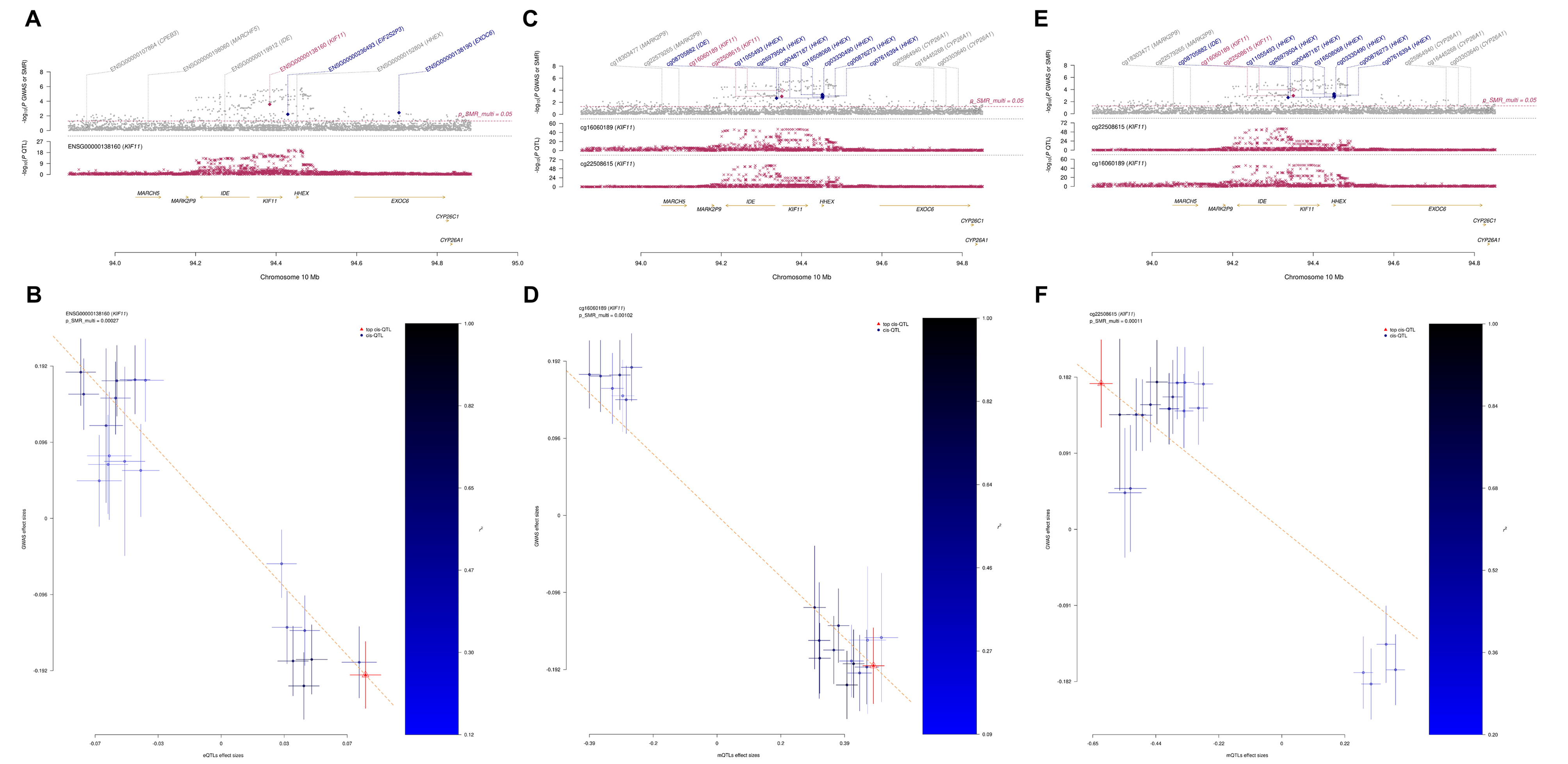

Supplement: Supplemental Material [file IRNF_A_2663248_SM8823.jpg]

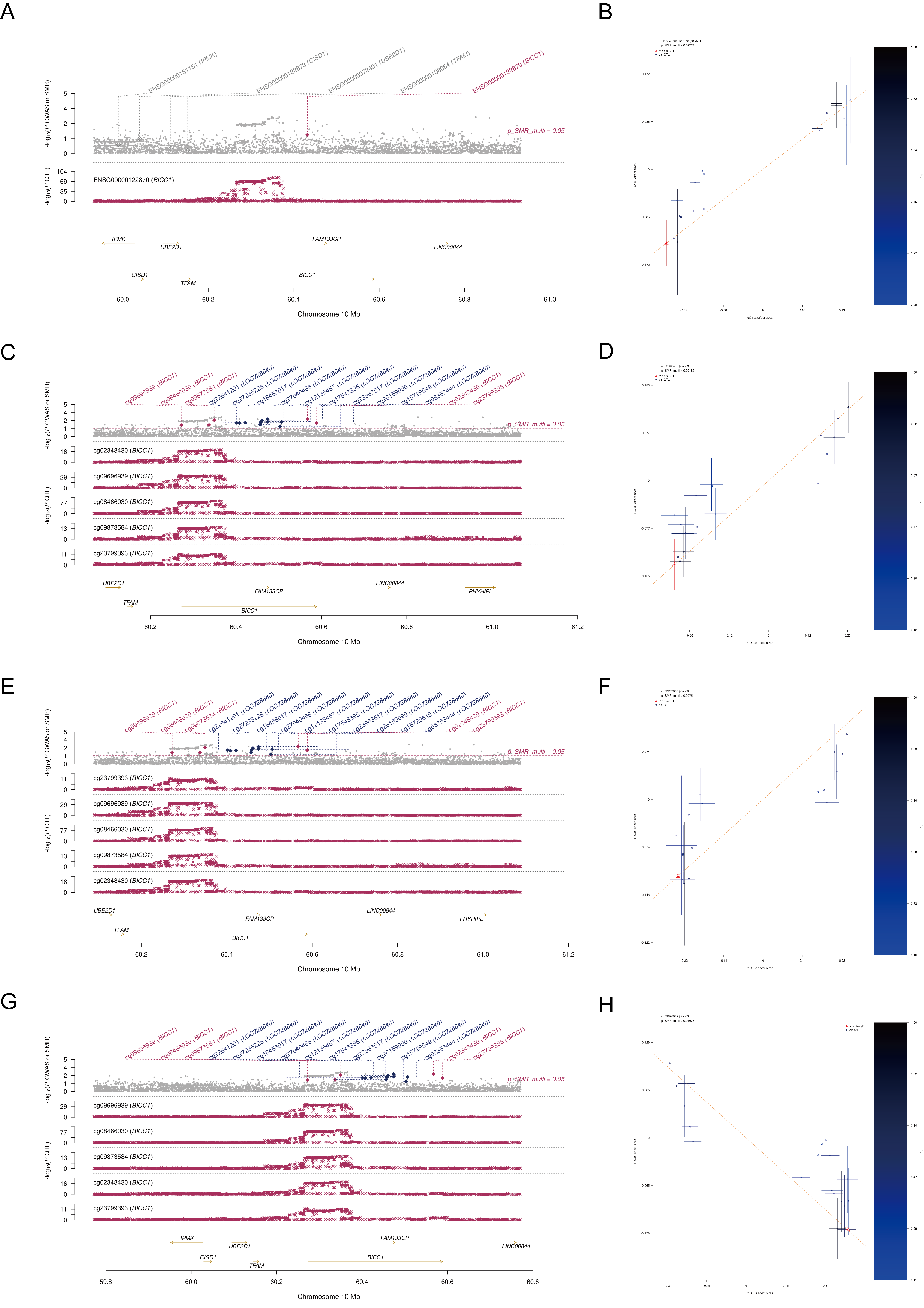

Supplement: Supplemental Material [file IRNF_A_2663248_SM8497.tif]

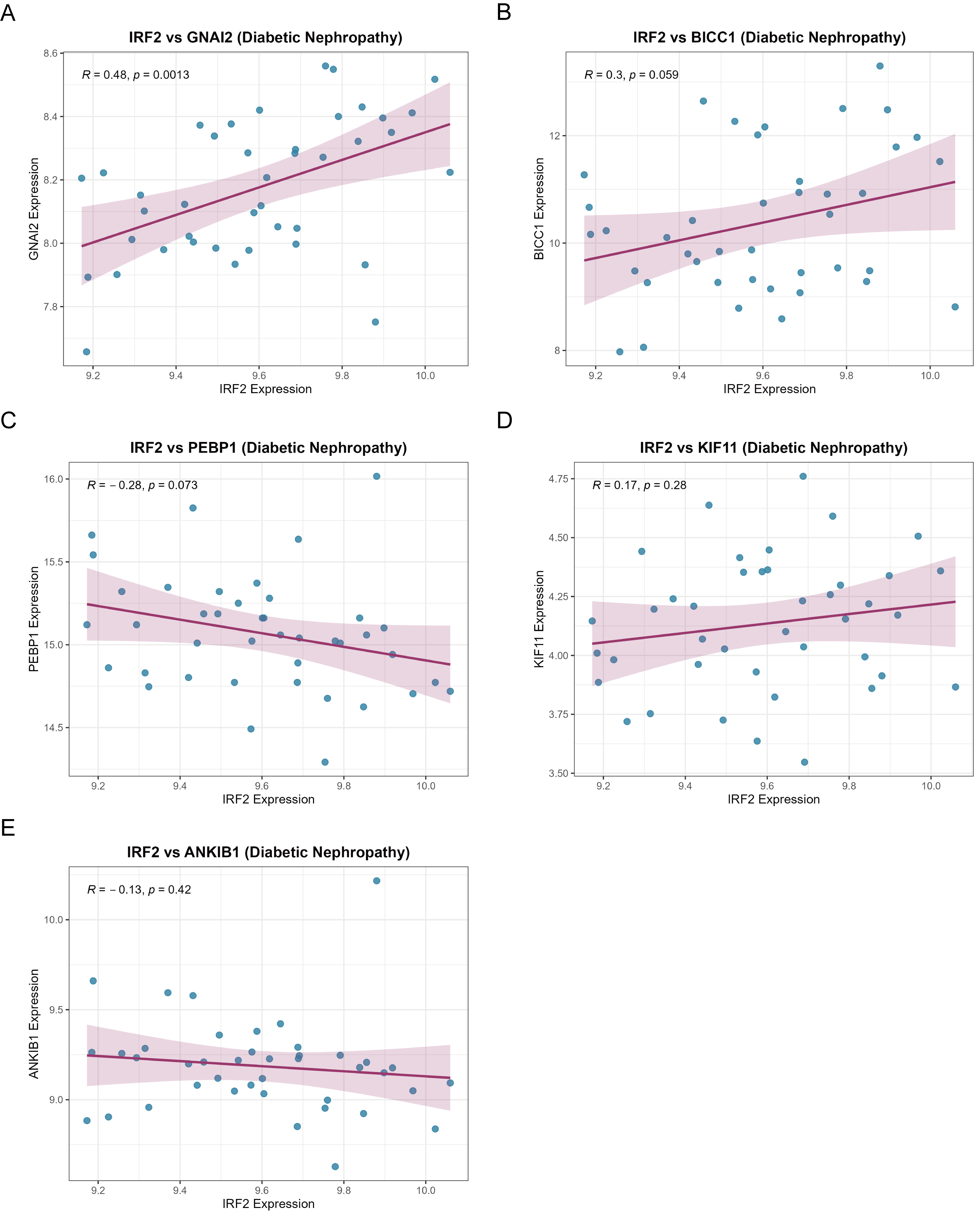

Supplement: Supplemental Material [file IRNF_A_2663248_SM8496.tif]

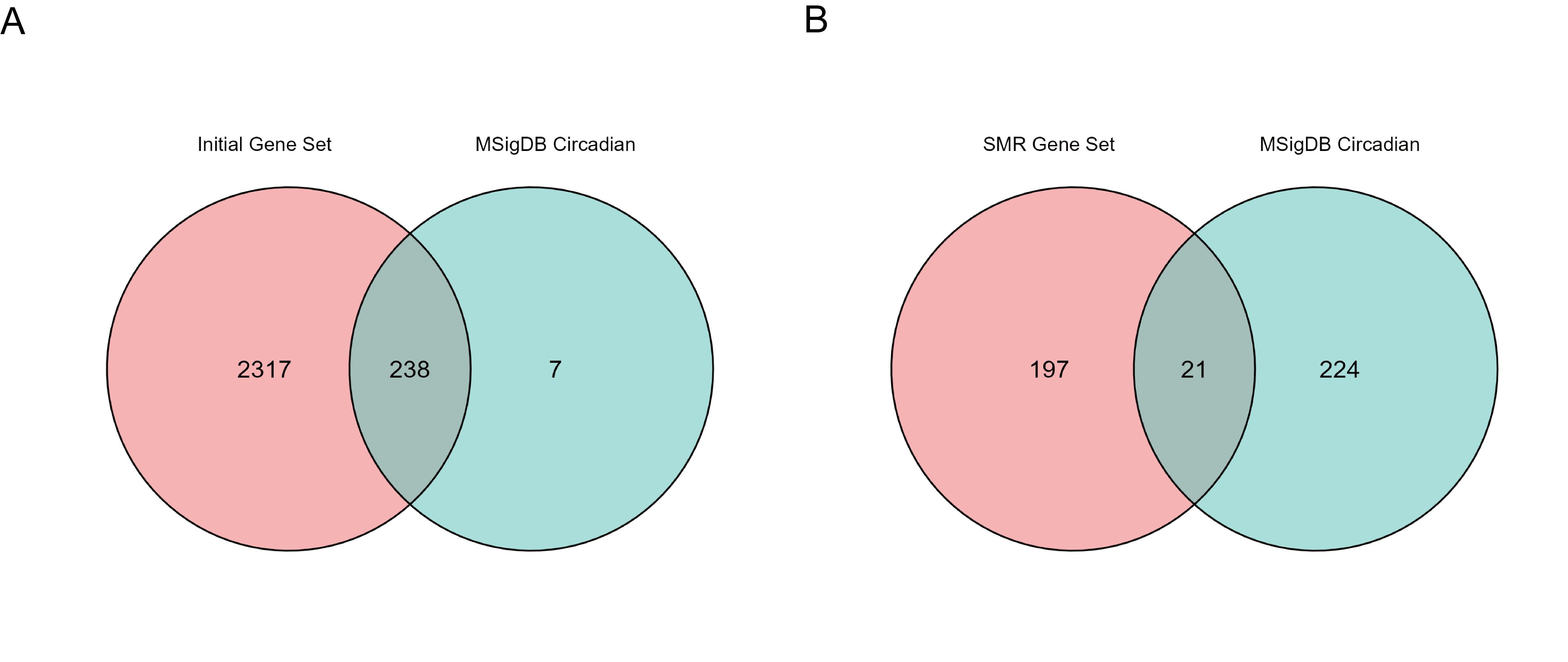

Supplement: Supplemental Material [file IRNF_A_2663248_SM8495.tif]

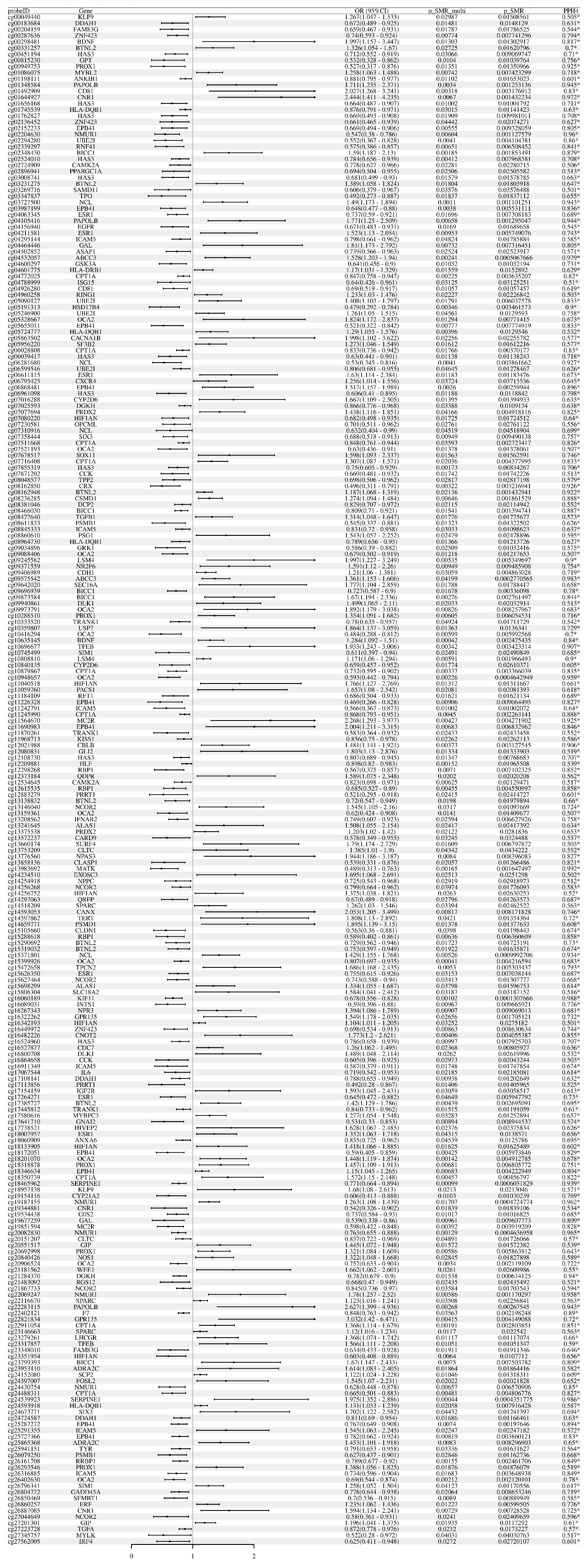

Supplement: Supplemental Material [file IRNF_A_2663248_SM8490.jpg]
